# Designing profitable, resource use efficient and environmentally sound cereal based systems for the Western Indo-Gangetic plains

**DOI:** 10.1038/s41598-020-76035-z

**Published:** 2020-11-06

**Authors:** Hanuman S. Jat, Virender Kumar, Ashim Datta, Madhu Choudhary, Yadvinder-Singh, Suresh K. Kakraliya, Tanuja Poonia, Andrew J. McDonald, Mangi L. Jat, Parbodh C. Sharma

**Affiliations:** 1 ICAR-Central Soil Salinity Research Institute (CSSRI), Karnal, India; 2 International Maize and Wheat Improvement Center (CIMMYT), New Delhi, India; 3 International Rice Research Institute (IRRI), Los Banos, Philippines; 4 Borlaug Institute for South Asia (BISA), Ludhiana, India; 5 Swami Keshwanand Rajasthan Agriculture University, Bikaner, India; 6 College of Agriculture and Life Sciences, Cornell University, Ithaca, NY USA

**Keywords:** Plant sciences, Ecology

## Abstract

In the western Indo-Gangetic plains, issues of deterioration in soil, water, and environment quality coupled with low profitability jeopardize the sustainability of the dominant rice–wheat (RW) system. To address these issues, crop diversification and conservation agriculture (CA)-based management hold considerable promise but the adoption of both approaches has been low, and additional evidence generation from a multi-criteria productivity and sustainability perspective is likely required to help drive the change. Compared to prevailing farmers’ practice (FP), results suggest that CA-based rice management increased profitability by 13% and energy use efficiency (EUE) by 21% while reducing irrigation by 19% and global warming potential (GWP) by 28%. By substituting CA-based maize for rice, similar mean profitability gains were realized (16%) but transformative improvements in irrigation (− 84%), EUE (+ 231%), and GWP (− 95%) were observed compared to FP. Inclusion of mungbean in the rotation (i.e. maize-wheat-mungbean) with CA-based management increased the system productivity, profitability, and EUE by 11, 25 and 103%, respectively while decreasing irrigation water use by 64% and GWP by 106% compared to FP. Despite considerable benefits from the CA-based maize-wheat system, adoption of maize is not widespread due to uneven market demand and assured price guarantees for rice.

## Introduction

In South Asia, cereal crop yields have grown remarkably since the 1960s due to intensive input use, modern crop genetics, and adoption of improved management practices. Nevertheless, aggregate production still must be increased by 60–70% over current levels to meet the expected food demand of the South Asian population (9.7 billion) by 2050^1^. The rice–wheat (RW) cropping system is the major cereal-based system for food, nutrition, and livelihood security in the Indo-Gangetic Plains (IGP) of South Asia, and is practiced on around 13.5 million ha^2^, contributing around half of the cereal production of India^3^. In South Asia, population growth, degradation of natural resources, and low factor productivity jeopardize both regional food security, and broader sustainable development goals. Continuous cultivation of RW with conventional tillage (CT) and traditional management practices coupled with residue burning has resulted in groundwater table depletion, high costs of cultivation and energy use, and deterioration in soil health and air quality in the western IGP^4–7^. Consequently, evidence suggests that the productivity of the RW system is either stagnating or declining. In North-west (NW) India, water tables declined at a rate of about 0.2 m year ^−1^ between 1973 and 2001, a trend that accelerated to 1.0 m year^−1^ between 2000 and 2006^8^. More recent data suggest declines on the order of 1.49 m in a single year in some locations^9^. Besides, evidence from NW India suggests that the traditional practice of soil puddling for rice reduces the yield of the following wheat by 12–15% due to its adverse effects on soil physical properties^4,10,11^. Furthermore, conventional RW systems may lead to depletion of soil organic carbon^12^.

Conservation agriculture (CA)-based crop management practices in the RW system has been done mostly on an individual crop basis (especially in wheat crop) to understand the effect of one or two practices/variables (tillage, residue management etc.) in the western IGP. But, under the growing complexity of expected climate change effects on agriculture would need the involvement of multiple management practices on system mode to tackle the issues of systems’ sustainability, and natural resources degradation. Substitution of CA-based management practices in single crop also helps in saving resources (water and energy) e.g. direct-seeded rice (DSR) instead of puddled transplanted rice (PTR)^4^ and sowing of maize on permanent beds (PB) instead of flat beds sowing. However, the adoption of DSR in the IGP of South Asia has been slow because of higher weed infestation, high incidence of iron deficiency^14^, and lack of suitable varieties^15^ that resulted lower rice yields^13^. Adoption of maize for replacing PTR is another potential alternative of RW systems in the western IGP^3,10,11,16^. Shrinking of the global trade for rice and the rising demand for maize from the poultry sector provides incentives for diversification away from rice towards maize^17^. In addition, cultivation of high yielding maize cultivars requires 80–85% less irrigation water compared to PTR in the IGP of South Asia^10,11,16^. The maize-wheat (MW) cropping system can potentially replace the rice from the RW system in some niches of the western IGP, especially in the areas where wheat experiences yield penalties due to delayed sowing because of late rice harvest^18,19^. In wheat-growing areas of NW India, Pathak et al.^20^ reported a yield loss of 15–60 kg ha^−1^ day^−1^ due to delayed sowing (beyond mid-November) and in that situation maize fits well as it matures by mid-October. Presently, cultivation of the MW system is practiced in about 1.86 million ha in the IGP^21^. In the monsoon season, water-logging is one of the major constraints to maize cultivation in the IGP, but some evidence suggests that it can be managed through CA-based management practices^10,11^.

Broad interest in CA is driven by its potential to conserve water and energy, and improving soil health while reducing greenhouse gas emissions^10,11^ against the conventional agricultural practices of cereal crop production^19,22^. Existing evidence from South Asia suggests that cultivation of rice/maize-wheat system on CA principles could help in enhancing the crop productivity and profitability^4,10,11,16,23^, sustaining soil health^24–30^, improving environmental quality^12,31–33^ and saving of irrigation water^11,16,23,34^. Integration of short-duration (60–65 days) pulse crop (mungbean) for sustainable intensification of cereal based systems with CT-based management practices could not be feasible for enhancing farm profitability, and nutritional security^16,19^. The information on energy use efficiency (EUE) related to different management scenarios and crop productivity is also one of the indicators to assess the systems’ performance^22^. In the last 2–3 decades, most efforts in IGP was rotated around zero-tillage (ZT) wheat in the RW system with limited emphasis on other crop management practices and cropping systems. To encash, the potential CA benefits, still the large knowledge gap exists related to the precise crop establishment, irrigation water and nutrient management on the performance of CA-based practices to scale out in the region.

Keeping the above facts in view, a study was conducted for 4-years to: (i) evaluate the impacts of CA-based management practices on crop yields, water productivity, energy use efficiency and profitability in RW and MW systems, (ii) identify optimal solution spaces with respect to yields, resource utilization, energy use efficiency, and global warming potential (GWP). We hypothesized that CA-practices (combination of ZT, PB, residue retention, crop diversification) would result in higher crop and water productivity with high net returns while improving the environmental quality compared with farmers’ practice of RW cultivation.

## Results

### Weather

All the weather parameters measured during the study period were similar to the long-term averages (Fig. S1). During the study period (2014–2018), crops received an average annual rainfall of 763 mm, although its distribution was quite different amongst the rainy season (June–September) (Fig. S1). Rice/maize season in 2014, 2015, and 2016, 2017 received 485 (256 mm in September), 420 (255 mm in July), 533 (284 mm in August), and 695 mm (247 mm in June and 226 mm in September) of rainfall, respectively. In 1st year, the wheat crop receivedrainfall of 247 mm whereas in the 2nd, 3rd, and 4th years it was only 56, 96 and 78 mm, respectively.

### Crops and system productivity

The management practices under different rice/maize-based scenarios influenced the crop grain yields over the 4-years (2014–2017) (Table 1). Scenarios with rice crops (Sc1-Sc3) did not differ in rice yields during the year 2014 and 2017, but CT direct seeded rice (Sc2) in the 2nd year (2015) and ZT direct seeded rice (Sc3) in the 3rd year (2016) produced 0.9 Mg ha^−1^ higher and 1.1 Mg ha^−1^ lower yield than farmers’ practice (Sc1), respectively (Table 1). Rice equivalent maize yields in CA-based scenarios (Sc6-Sc7) did not differ from scenarios with rice crops (Sc1-Sc3) in any of the study years. Rice equivalent maize yield of CA-based Sc5 with maize on PB, although was similar to Sc1 in all the years but was 1.41 Mg ha^−1^ lower than ZT-DSR (Sc3) in 1st year and 0.98 Mg ha^−1^ lower than CT-DSR (Sc2) in 2nd year. In contrast, rice equivalent yield (REY) of Sc4 with maize on fresh beds (FB) produced lower yields than one of the rice-based scenarios in three out of four years. These results suggest that maize performs better under CA-based management system than under conventional tillage system. Almost 5% higher yield of maize was recorded in the 1st year and 12–16% higher in the last three years under CA-based scenario (Sc7) compared to CT-based scenario (Sc4) and at par with Sc5. Based on the 4-years average, rice equivalent yield (REY) of Sc4 (maize on FB ) was 0.8 Mg ha^−1^ (12%) lower than Sc1 (business-as-usual) whereas other scenarios did not differ from each other in REY (Table 1).

**Table 1:** Effect of different scenarios on grain yields (Mg ha^−1^) of rice, maize, wheat and systems during 4-years (2014–2018).

Scenarios^a^	2014–2015			2015–2016			2016–2017			2017–2018			4-yr mean		
	Rice/maize	Wheat	System	Rice/maize	Wheat	System	Rice/maize	Wheat	System	Rice/maize	Wheat	System	Rice/maize	Wheat	System
Sc1	6.85^ABC^^b^	5.27^AB^	12.47^BC^	5.58^BC^	5.26^AB^	11.27^BC^	6.87^A^	5.46^B^	12.91^B^	6.57^A^	5.40^B^	12.62^A^	6.47^A^	5.35^B^	12.32^B^
Sc2	7.33^AB^	5.52^A^	13.22^AB^	6.45^A^	5.76^A^	12.68^AB^	6.10^AB^	6.24^A^	13.00^B^	6.10^AB^	5.86^AB^	12.66^A^	6.50^A^	5.84^A^	12.89^B^
Sc3	7.70^A^	5.52^A^	13.58^A^	5.82^AB^	5.19^AB^	11.43^AB^	5.74^B^	5.99^AB^	12.37^B^	6.14^AB^	5.75^AB^	12.58^AB^	6.35^A^	5.61^AB^	12.49^B^
Sc4	6.39^BC^ (6.39)*	5.44^AB^	12.18^C^	4.88^C^(5.19)	4.64^B^	9.89^C^	6.04^AB^ (6.50)	5.69^AB^	12.32^B^	5.42^B^ (5.90)	5.50^AB^	11.14^B^	5.68^B^ (6.05)	5.31^B^	11.38^C^
Sc5	6.29^BC^ (6.29)	5.03^B^	11.66^C^	5.47^BC^ (5.82)	5.84^A^	11.78^AB^	6.90^A^ (7.43)	6.20^A^	13.75^AB^	6.61^A^ (7.19)	6.10^A^	12.90^A^	6.32^A^ (6.74)	5.79^A^	12.52^B^
Sc6	6.47^C^ (6.47)	5.31^AB^	11.90^C^	5.67^ABC^(6.03)	5.34^AB^	11.44^AB^	6.14^AB^ (6.62)	6.04^AB^	12.81^B^	6.56^A^ (7.14)	5.93^AB^	12.67^A^	6.15^AB^ (6.56)	5.65^AB^	12.21^B^
Sc7	6.94^ABC^ (6.94)	5.35^AB^	13.57 ^A^ (0.35)**	5.71^AB^ (6.08)	5.57^AB^	12.76^A^(0.30)**	6.81^AB^ (7.33)	6.33^A^	14.84^A^ (0.29)**	6.23^AB^ (6.78)	5.88^AB^	13.36^A^ (0.29)**	6.36^A^ (6.78)	5.78^A^	13.63^A^ (0.31)**

^a^Refer Table 4f or scenarios description.

^b^Means followed by a similar uppercase letters within a column are not significantly different at 0.05 level of probability using Tukey’s HSD test.

^c^System grain yield was expressed as rice-equivalent yield (t ha^−1^).

*Maize yield in parenthesis.

**Mungbean yield in parenthesis.

The management practices influenced wheat grain yield over the years of experimentation (Table 1). Across study years, the grain yield of ZT wheat in CA-based scenario was either similar or higher than CT wheat. Results showed significantly (*P* < 0.05) higher wheat grain yield in all CA-based scenarios (Sc2-Sc3, and Sc5-Sc7) compared to CT (Sc1 and Sc4). CA-based scenarios produced a ~ 9% higher wheat grain yield compared to farmers’ practice (FP; Sc1). Almost similar yield of wheat was recorded with CA-based management whether it was grown after rice or maize.

System yield (rice equivalent yield; REY) varied from 9.89 to 14.84 Mg ha^−1^ over the study years (Table 1). Four-year mean system yield (rice equivalent) of CA-based Sc7 was 0.74 to 2.25 Mg ha^−1^ (6–20%) higher than rest of the scenarios. The lowest system yield was recorded in Sc4 with maize-wheat on a FB with a 17% lower yield than Sc7, and 7–12% lower than the rest of the scenarios. System-level yield of Sc7 was consistently highest in all the study years, whereas Sc4 had the lowest yield. In terms of system productivity, among different practices, Sc2 (+ 5%) and Sc7 (+ 11%) were the most efficient management practices in the RW system and MW system, respectively.

### Sustainable yield index (SYI)

The sustainable yield index (SYI) for rice, maize, wheat, and system are presented in Fig. S2. Highest SYI for rice/maize was observed under Sc2 (0.81) and Sc7 (0.81), while the lowest with CT-based maize system (Sc4). SYI for wheat was higher for CA-based management scenarios (Sc2, Sc5, and Sc7) (0.83–0.84) compared to CT-based scenarios (Sc1 and Sc4). Results indicated that wheat yields are more sustainable as compared to rice and maize. Compared to farmers’ practice, SYI was increased by 11 and 5% in Sc7 and Sc2, respectively. Results from our study clearly showed that CA-based Sc7 (maize-wheat-mungbean) is more sustainable than that of the other rice/maize-based scenarios.

### Economic profitability

Crop production costs were mainly attributed to tillage/field preparation, crop establishment, field preparations, irrigation, fertilizer, pest management, harvesting/threshing, and man-days involved in agricultural production. The total production costs of rice and maize varied from 541 to 715 USD ha^−1^ acros 4-years under different management scenarios (Table S1). Average (4-years’ mean) production costs of rice/maize was highest in CT-based rice (680 USD ha^−1^) and followed by CT-based maize(630 USD ha^−1^)and were lower (583-613USD ha^−1^) in CA-based management scenarios (Sc2-Sc3 and Sc5-Sc7) (Table S1). Compared to Sc1, the total production cost was ~ 13% lower when rice was seeded under ZT and maize on PB (permanent beds) (Table S1). In contrast, net income was highest in CA-based Sc5 (991 USD ha^−1^) followed by Sc7 (985 USD ha^−1^), and was lowest in Sc4 (741 USD ha^−1^) (Table 2). The net income of other CA-based scenarios (Sc2, Sc3, and Sc6) did not differ from Sc5 and Sc7. The net income of CA-based Sc5, Sc7, and Sc3 were 19, 18, and 12% higher, respectively compared to the CT-based RW system (835 USD ha^−1^) (Table 2).

**Table 2:** Effect of different scenarios onnet returns, water use, water productivity and energy use efficiency of rice, maize, wheat and systems (based on 4-years’ mean, 2014–2018.

Scenarios^a^	Net returns (USD ha^−1^)			Irrigation water use (mm ha^−1^)			Irrigation water productivity (kg grain m^−3^)			Energy use efficiency (MJ^−1^ MJ^−1^)		
	Rice/maize	Wheat	System	Rice/maize	Wheat	System	Rice/maize	Wheat	System	Rice/maize	Wheat	System
Sc1	835^BCb^	974^C^	1810^C^	2173^A^	454^A^	2627^A^	0.30^C^	1.21^C^	0.42^C^	3.95^C^	7.44^C^	5.05^E^
Sc2	946^AB^	1192^A^	2138^AB^	1759^B^	448^A^	2207^B^	0.39^C^	1.34^B^	0.52^C^	4.70^C^	9.65^AB^	6.23^D^
Sc3	945^AB^	1125^AB^	2070^B^	1753^B^	458^A^	2211^B^	0.38^C^	1.28^BC^	0.51^C^	4.85^C^	9.26^B^	6.25^D^
Sc4	741^C^	1005^BC^	1747^C^	316^C^	448^A^	764^CD^	2.25^AB^	1.23^BC^	2.23^AB^	10.81^B^	7.84^C^	9.25^C^
Sc5	991^A^	1167^A^	2158^AB^	289^C^	403^B^	692^D^	2.59^A^	1.48^A^	2.56^A^	13.82^A^	10.05^A^	11.92^A^
Sc6	935^AB^	1140^A^	2075^B^	359^C^	456^A^	815^CD^	2.06^B^	1.29^BC^	2.09^B^	12.68^A^	9.27^B^	10.95^B^
Sc7	985^A^	1175^A^	2261^A^	365^C^	451^A^	953^C^	2.15^B^	1.32^BC^	2.09^B^	12.72^A^	9.51^AB^	10.26^B^

^a^Refer Table 4 for scenarios description.

^b^Means followed by a similar uppercase letters within a column are not significantly different at 0.05 level of probability using Tukey’s HSD test.

In the case of wheat, based on a 4-year average, the cultivation cost and net returns varied from 456 to 534 USD ha^−1^ and 974 to 1192USD ha^−1^, respectively (Table S1 and Table 2). Similarly to rice and maize, CT-based management practices (Sc1-USD 534 ha^−1^ and Sc4-USD 495 ha^−1^) recorded the highest cost of wheat cultivation (Table S1) and CA-based scenarios recorded the lowest cultivation cost of USD 461 ha^−1^. Net income from wheat under CA-based management (Sc2, Sc3 and Sc5) was higher by 151–218 USD ha^−1^ (+ 16–22%) compared to Sc1 (974 USD ha^−1^) (Table 2).

**Figure 1:**
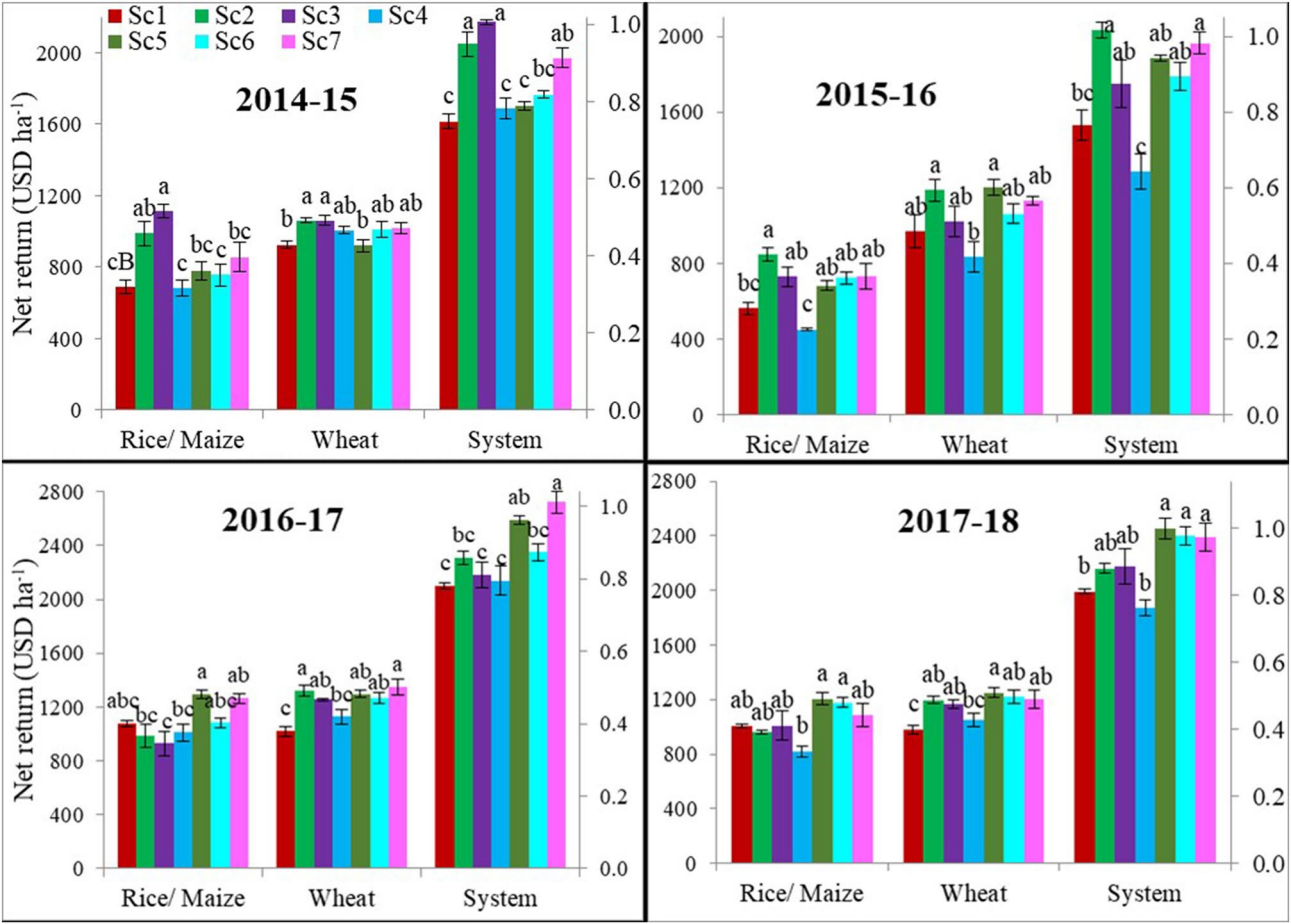
Effect of different scenarios on net returns (USD ha^−1^) of rice, maize, wheat and systems during 4-years (2014–18).

The total cultivation cost and net returns ranged from 988 to 1290 USD ha^−1^ and 1286 to 2592 USD ha^−1^, respectively under different system based management scenarios over the years (Table S1 and Fig. 1). On 4-year average basis, the highest cost of cultivation was associated with Sc1 (1213 USD ha^−1^) followed by Sc7 (1184 USD ha^−1^) and Sc4 (1124 USD ha^−1^) and, it was lowest with Sc3 (USD 1044 ha^−1^) (Table S1). The net incomes of all CA-based scenarios were higher than CT-based scenarios (Sc1 and Sc4) by 260–514 USD ha^−1^. CA-based Sc2, Sc3, Sc5, Sc6 and Sc7 recorded 18, 14, 19, 15 and 25% (4-years’ mean) higher net incomes, respectively compared to farmers’ practice (1810 USD ha^−1^) (Table 2). CA-based Sc2 (+ 18%) under RW system and CA-based Sc7 (+ 25%) under MW system, were the most profitable management scenarios compared to Sc1 among all the management scenarios included in the study (Table 2).

### Irrigation water use and water productivity

**Figure 2:**
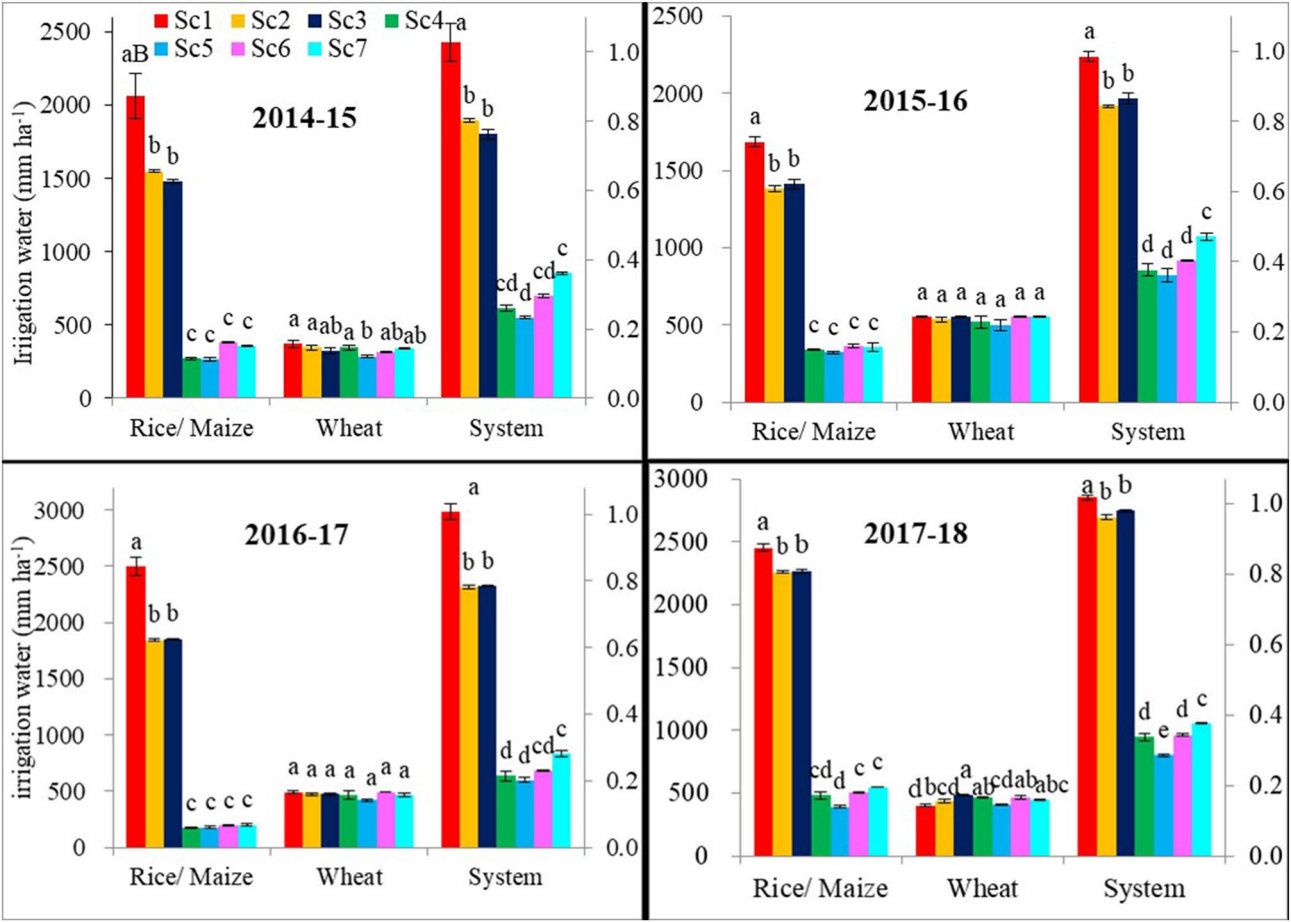
Effect of different scenarios on water use (mm ha^−1^) under rice, maize, wheat and systems during 4-years (2014–18).

The amount of irrigation water applied varied from 1382 to 2495 mm ha^−1^ in rice and 173 to 545 mm ha^−1^ in maize over the 4-years (Fig. 2). Based on 4-year average, the irrigation water input decreased in the following order: Sc1 (2173 mm ha^−1^) > Sc2 = Sc3 (1753–1759 mm ha^−1^) > S7 = Sc6 = Sc4 = S5 (289–365 mm ha^−1^) (Table 2). The same trend followed in all the study years except in the 4th year, where irrigation water input in Sc5 (maize on PB) was 109–154 mm ha^−1^ (22–28%) lower than Sc6 and Sc7 (ZT maize on flat beds). The amount of water applied in CT-based rice crop (Sc1; farmers’ practice) was significantly (P < 0.05) higher by ~ 19 and 85% (4-years’ mean) compared to CA-based rice (Sc2-Sc3) and maize (Sc5-Sc7) scenarios, respectively (Table 2). However, compared to CA-based rice (Sc2-Sc3), CA-based maize (Sc5-Sc7) saved ~ 79% of irrigation water. In the case of wheat, applied irrigation water varied from 285 to 555 mm ha^−1^ across the 4-years (Fig. 2).

In wheat, the amount of irrigation water applied was almost similar across the different scenarios except in Sc5 (Fig. 2), where about 12% (4-years’ mean) less irrigation water was applied compared to CT-based Sc1(Table 2). Based on 4-year average, scenarios did not differ in irrigation inputs during wheat except Sc5 which had 45–55 mm ha^−1^ (10–12%) lower irrigation input than rest of the scenarios (Table 2). At system level, the amount of applied water was significantly lowered by 16% (4-years’ mean) in CA-based rice systems (Sc2-Sc3) and by 70% (4-years’ mean) in maize-based systems (Sc4-Sc7), irrespective of management systems compared to CT-based RW system (2627 mm ha^−1^). The general trend in irrigation water input in different scenarios across years and average of four-years followed the following trend: Sc1 > Sc2 = Sc3 > Sc7 > Sc4-Sc6.

**Figure 3:**
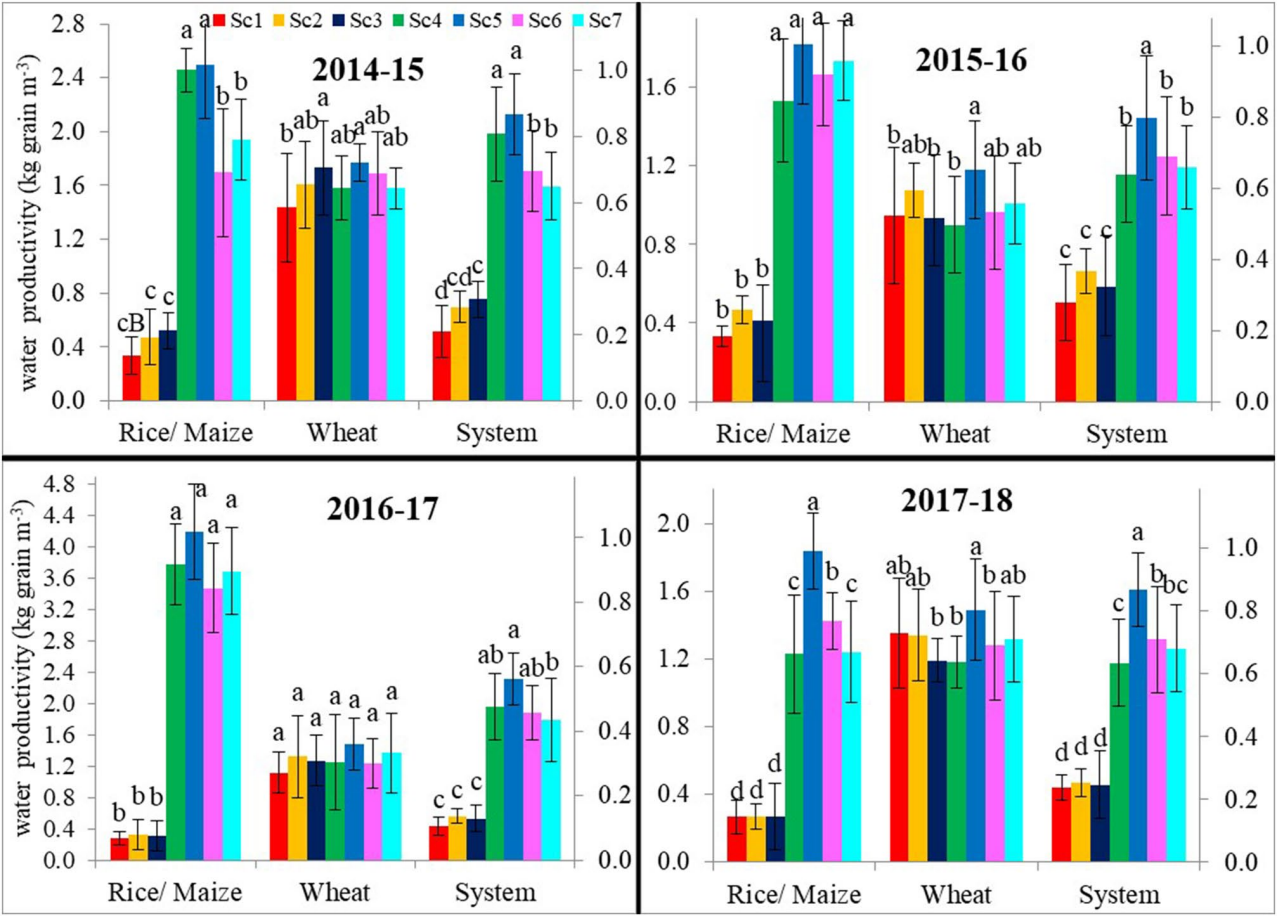
Effect of different scenarios on irrigation water productivity (kg grain m^−3^) of rice, maize, wheat and systems during 4-years (2014–2018).

Higher grain yield and low water use led to significantly (P < 0.05) higher irrigation water productivity (WP_I_) under CA-based management systems in all the crops and cropping systems compared to CT-based scenario (Sc1) (Fig. 3). On 4-year average basis, CA-based rice (Sc2-Sc3) and maize (Sc5-Sc7) recorded ~ 27 and 664% higher WP_I_ compared to CT-based Sc1 (0.42 kg grain m^−3^) (Table 2). On 4-year average basis, mean WP_I_ in maize was 583, 612, 644 and 755% higher in order of Sc5 (2.59 kg grain m^−3^) > Sc4 (2.25 kg grain m^−3^) > Sc7 (2.15 kg grain m^−3^) > Sc6 (2.06 kg grain m^−3^), respectively compared to Sc1 (0.30 kg grain m^−3^) (Table 2). In wheat, CA-based management practices increased WP_I_ by 9% (4-years’ mean) compared to Sc1 (1.21 kg grain m^−3^). CA-based management practices improved mean WP_I_ by 23 and 438% in RW and MW system, respectively compared to Sc1 (0.42 kg grain m^−3^).

### Energy use efficiency

**Figure 4:**
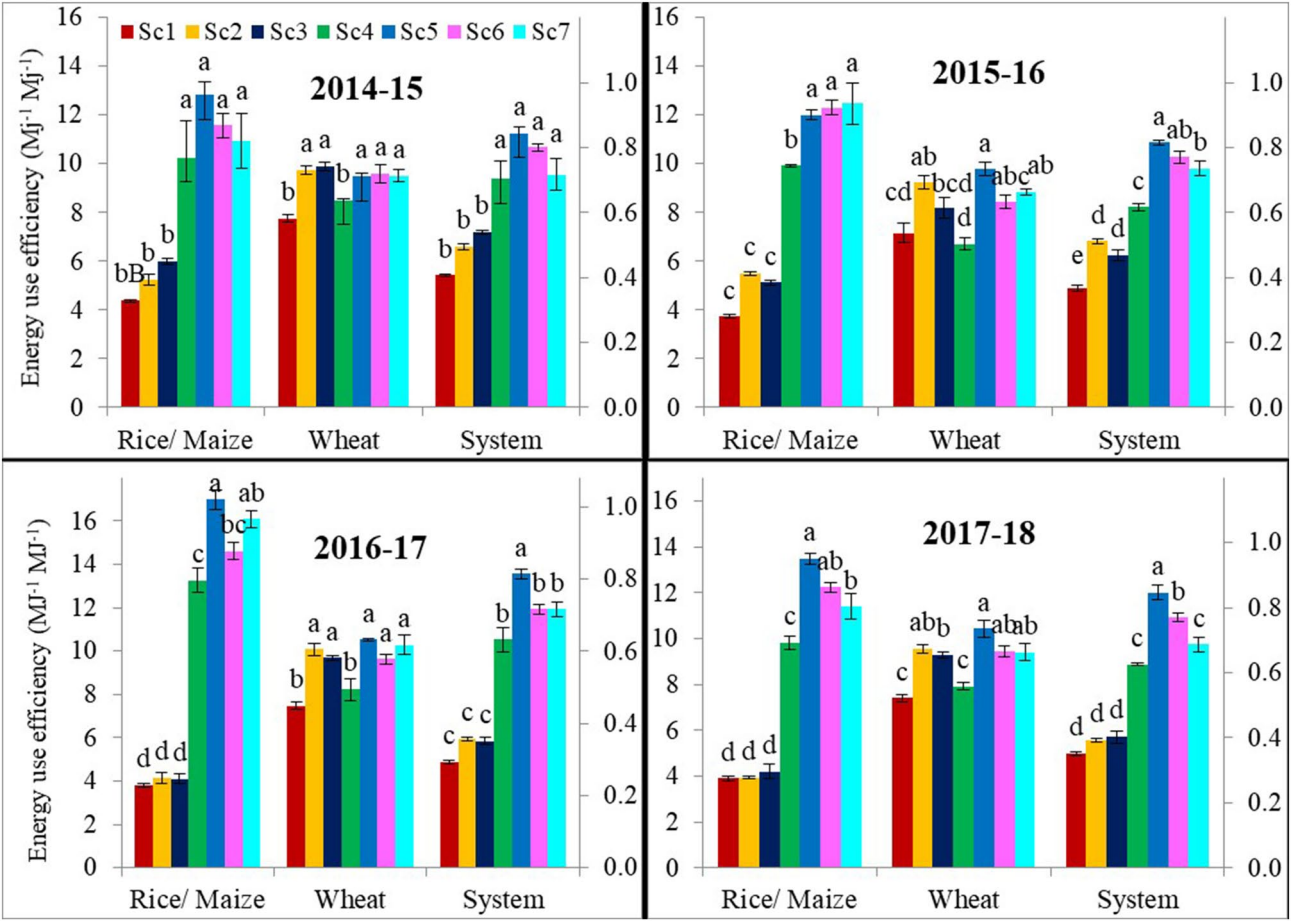
Effect of different scenarios on energy use efficiency of rice, maize, wheat and systems during 4-years (2014–2018).

Energy equivalents for different agricultural operations used in the study are given in Table S2. The energy input and output (Tables S3 and S4), and energy use efficiency (EUE) of rice, maize, wheat and mungbean were influenced by the management practices and varied from year to year (Fig. 4). During rice/maize, higher EUE was observed in maize based scenarios (Sc4-Sc7) than in rice-based scenarios (Sc1-Sc3) (10.81–13.83 MJ MJ^−1^ versus 3.95–4.85 MJ MJ^−1^) (Table 2). Rice-based scenarios (Sc1-Sc3) did not differ in EUE. However, in maize-based scenarios (Sc4-Sc7), EUE of CA-based maize scenarios (Sc5-Sc7) was 17–28% higher than CT-based maize Sc4. Across years also, the same trend was observed with no difference in EUE of rice-based scenarios (Sc1-Sc3), whereas CA-based maize scenarios (Sc5-Sc7) had higher EUE than CT-based Sc4 (Table 2). In wheat crop, highest EUE was observed under CA-based scenarios (Sc2-Sc3 and Sc5-Sc7) compared to CT-based scenarios (Sc1 and Sc4) across all study years and based on four years’ average (9.26–10.05 MJ MJ^−1^ versus 7.44–7.84 MJ MJ^−1^), it is indicated that CA-based scenarios are more energy-efficient than those of CT-based scenarios (Fig. 4). In all the years, EUE of maize-based scenarios (Sc4-Sc7) were higher than rice-based scenarios (Sc1-Sc3) but within rice-based scenarios (Sc1-Sc3), results were more variable with higher EUE of CA-based Sc2 and Sc3 in 1st and 2nd year than CT-based scenarios (Sc1) but did not differ in 3rd and 4th year (Fig. 4). On system basis, the EUE of different scenarios decreased in the following order: Sc5 (11.92 MJ MJ^−1^) > Sc6 = Sc7 (10.26–10.95 MJ MJ^−1^) > Sc4 (9.25 MJ MJ^−1^) > Sc3 = Sc2 (6.23–6.25 MJ MJ^−1^) > Sc1 (5.05 MJ MJ^−1^) (Table 2). Maize-based scenarios (Sc5-Sc7) had 48 to 136% higher EUE than rice-based scenarios (Sc1-Sc3) suggesting maize-wheat based cropping systems were more efficient in energy use than rice–wheat based systems (Table 2). Scenario 3 (+ 24%) in RW and Sc5 (+ 136%) in MW system were the most energy-efficient among the different combinations of management practices in 4-years of study.

### Methane (CH_4_) and nitrous oxide (N_2_O) emission from soil

Methane (CH_4_) was emitted only from the rice plots (Table 3). The estimated mean value of CH_4_ emission (kg CO_2_ eq. ha^−1^) was 39% lower in CA-based rice scenarios without continuous flooding (Sc2 and Sc3) compared to CT-based Sc1 with continuous flooding for > 1 month (Table 3).

**Table 3:** Effect of different scenarios on GHGs emissions, C-sequestration and GWP of rice, maize, wheat and systems (based on 4-year average, 2014–18).

Scenarios^a^	CH_4_ kg (kg CO_2_ eq. ha^−1^)	N_2_O (kg CO_2_eq. ha^−1^)	GHG emission due to residue burning (kg CO_2_eq. ha^−1^)	GHG emission due to energy consumption (kg CO_2_eq. ha^−1^)	Total C sequestration (kg CO_2_ eq. ha^−1^)	Area Scaled (GWP; kg CO_2_eq. ha^−1^)
**Rice/maize**						
Sc1	1818	7	278	2941^A^	0	5043^A^
Sc2	1103	583	0	2484^B^	− 428	3742^B^
Sc3	1129	580	0	2414^B^	− 625	3498^B^
Sc4	0	50	69	1126^C^	0	1245^C^
Sc5	0	60	0	1005^D^	− 851	213^D^
Sc6	0	59	0	1091^CD^	− 866	285^D^
Sc7	0	61	0	1097^CD^	− 908	250^D^
**Wheat**						
Sc1	0	50	59	1299^A^	0	1409^A^
Sc2	0	101	0	1174^B^	− 1658	− 384^C^
Sc3	0	102	0	1183^B^	− 1821	− 536^C^
Sc4	0	50	58	1298^A^	0	1407^A^
Sc5	0	72	0	1122^C^	− 1179	16^B^
Sc6	0	72	0	1183^B^	− 1204	51^B^
Sc7	0	73	0	1178^B^	− 1243	8^B^
**Rice/maize-wheat system**						
Sc1	1818	57	337	4240^A^	0	6451^A^
Sc2	1103	683	0	3658^B^	− 2086	3359^B^
Sc3	1129	682	0	3597^B^	− 2446	2962^BC^
Sc4	0	101	109	2424^C^	0	2652^C^
Sc5	0	132	0	2127^E^	− 2030	228^D^
Sc6	0	131	0	2274^D^	− 2070	336^D^
Sc7	0	171	0	2435^C^	− 3039	− 433^E^

^a^Refer Table 4 for scenarios description*Included diesel, electricity, and production and transportation of fertilizers.

N_2_O emission varied from 7 to 583 kg CO_2_ eq. ha^−1^ during the rice season (Table 3). The maximum amount of N_2_O emission (580–583 kg CO_2_ eq. ha^−1^) was observed in CA-based rice scenarios (Sc2-Sc3) followed by the maize-based scenarios (50–61 kg CO_2_ eq. ha^−1^) and was the lowest in CT-based rice Sc1 (7 kg CO_2_ eq. ha^−1^). The CA-based rice and maize scenarios produced 88 and 9 times higher N_2_O emission compared to Sc1, respectively. The N_2_O emission in the wheat season ranged between 50 to 102 kg CO_2_ eq. ha^−1^ (Table 3). The highest N_2_O emission was estimated with CA-based scenarios (Sc2-Sc3) (101–102 kg CO_2_ eq. ha^−1^) and followed by scenarios Sc5-Sc7 (72–73 kg CO_2_ eq. ha^−1^) and was lowest in CT-based scenarios Sc1 and Sc4 (50 kg CO_2_ eq. ha^−1^). The N_2_O emission in the wheat crop was increased by 57% under CA-based management scenarios compared to CT-based management scenario (Table 3). On system basis, CA-based rice and maize systems emitted 12 and 2.4 times more N_2_O compared to Sc1, respectively but methane emission was reduced to zero (Table 3). Overall CA-based cereal management systems emitted almost six-time higher N_2_O emission compared to farmers’ practice, irrespective of cropping systems (Table 3).

### GHG emission associated with residue burning (kg CO_2_ eq. ha^−1^)

Crop residue burning is a common farmers’ practice in the western IGP. Therefore, GHG emission due to residue burning (kg CO_2_ eq. ha^−1^) was estimated with CT-based system of rice (Sc1; 278 kg CO_2_ eq. ha^−1^) and maize (Sc4; 69 kg CO_2_ eq. ha^−1^) cultivation (Table 3). In the case of wheat, the GHG emission due to residue burning (kg CO_2_ eq. ha^−1^) was estimated with CT-based cultivation of wheat in Sc1 (59 kg CO_2_ eq. ha^−1^) and Sc4 (40 kg CO_2_ eq. ha^−1^). No GHG emission (kg CO_2_ eq. ha^−1^) was considered due to burning where crop residues were retained/incorporated in CA-based management practices under different scenarios.

### GHG emission due to energy consumption (kg CO_2_ eq. ha^−1^)

GHG emission due to energy consumption varied from 2414 to 2941, 1005 to 1126 and 1122 to 1299 kg CO_2_ eq. ha^−1^ in rice, maize, and wheat, respectively (Table 3). Compared to CA-based management scenarios, CT-based scenarios emitted more GHGs due to the higher consumption of electricity and diesel energy in all the crops and cropping systems. Compared to Sc1, GHG emission due to energy consumption from rice/maize season was 16–18% lower in CA-based rice scenarios (Sc2-Sc3) and 63–66% lower in maize-based scenarios (Sc4-Sc7) (Table 3). Overall, compared to Sc1, CA-based scenarios reduced ~ 17 and 63% of GHG emissions due to energy consumption in rice and maize across the years, respectively. Similarly, in wheat, CA-based scenarios (Sc2-Sc3 and Sc5-Sc7) reduced 10% GHG emission due to energy consumptions as compared to CT-based scenarios (Sc1 and Sc4). On the system basis, Sc2, Sc3, Sc4, Sc5, Sc6, and Sc7 recorded lower energy-related emission of GHG by 14, 15, 43, 50, 46, and 43% (4-years’ mean), respectively, relative to Sc1 (4240 kg CO_2_ eq. ha^−1^) (Table 3). Rice and maize-based systems recorded ~ 15 and 46% lower GHG related emissions, respectively compared to farmers’ practice (Sc1-4240 kg CO_2_ eq. ha^−1^).

### Carbon (C) sequestration

The estimated C-sequestration was carried out in those scenarios where crop residues were retained/ incorporated during the study period. The C-sequestration varied with the amount of crop residue was recycled under different crops and cropping systems. Estimated C-sequestration in soil varied from 0 to − 625 kg CO_2_ eq. ha^−1^ in rice, 0 to − 908 CO_2_ eq. ha^−1^ in maize and 0 to − 1821 kg CO_2_ eq. ha^−1^ in wheat (Table 3). On system basis, the highest C-sequestration was estimated under CA-based management scenarios which varied in the following order of Sc7 (3039 kg CO_2_ eq. ha^−1^) > Sc3 (2446 kg CO_2_ eq. ha^−1^) > Sc2 (2086 kg CO_2_ ha^−1^) > Sc6 (2070 kg CO_2_ eq. ha^−1^).

### Total global warming potential (GWP)

Global warming potential (GWP) varied with crop management practices under different scenarios over the years. In 4-year, the total estimated GWP from rice was lower under the CA-based systems than CT-based system. On 4-year mean basis, the GWP under the CA-based rice (Sc2-Sc3) and maize (Sc5-Sc7) systems were lowered by ~ 28 and 90% compared to farmers’ practice (Sc1), respectively (Table 3). Within maize-based scenarios, the CA-based scenarios (Sc5-Sc7) reduced the GWP of maize by 77–83% compared to CT-based Sc4. The GWP in wheat varied from − 384 to 1409 kg CO_2_ eq. ha^−1^ based on 4 year average (Table 3). The 4 years mean GWP was significantly lower by 127–138% in CA-based RW system (Sc2-Sc3) and 96–99% in CA-based MW system (Sc5-Sc7) compared to Sc1, respectively (Table 3). The mean GWP of wheat under CT-based RW system (Sc1) was similar to CT-based MW (Sc1and Sc4) systems.

The crop management practices under different scenarios influenced the total GWP (CO_2_ eq. ha^−1^) in both the cropping systems (RW and MW system) during the study years (Table 3). On 4-years system mean basis, GWP under Sc2, Sc3, Sc4, Sc5, Sc6, and Sc7 were 48, 54, 59, 96, 95, and 107% lower compared to Sc1 (farmers’ practice), respectively. In CA-based RW and MW systems, GWP was estimated lower by 50 and 89% compared to CT-based Sc1(6451 kg CO_2_ eq. ha^−1^), respectively.

## Discussion

Rice yield was not much changed with different agronomic management practices over the first 2-years. However, the yield of both CT and ZT-DSR declined over CT rice (Sc1) after 2 years of experimentation. These results align with the findings of Kreye et al*.*^14^ and Peng et al*.*^35^ who found that multi-micronutrient deficiencies and nematode infestation increases over time in DSR, resulting in yield declines compared to puddled transplanted rice (PTR). Kumar et al*.*^11^ also reported lower yields in ZT-DSR compared to transplanted rice under similar ecologies due to the unavailability of suitable aerobic rice cultivar and occurrence of iron deficiency. The results of our study showed higher yields of maize under CA-based management systems compared to CT-based systems in all the years, irrespective of planting on flat beds and on PBs, and increased to 12–16%. Consistent with our results, higher maize yield under ZT/PBs compared to planting on the flat beds or on FB was also reported in several studies^23,34,36^. Compared to CT maize (Sc4), higher maize yield in CA-based scenarios was probably due to favorable soil temperature and moisture conditions created by residue mulch and efficient use of irrigation waterand nutrients^23,34^. Rashid et al*.*^37^ reported 32% higher yield of maize with 50% straw retention compared to straw removal. The results from our study showed 5–9% higher wheat grain yield in all CA-based scenarios compared to CT-based system (Sc1 and Sc4). The higher yields in all CA-based scenarios for wheat are likely caused by the combined effect of early sowing date (last week of October versus second fortnight of November), improved soil health conditions under CA-based systems through crop residue retention and legume integration. In the IGP region, many studies have shown that growing rice without puddling (e.g. with DSR) has beneficial effects on the succeeding wheat crop by avoiding soil compaction^4,10,11^. CA-based management in cereal systems improved the soil physical and chemical properties^28–30,38^, and biological properties^24–27^. These improved soil conditions led to better germination, crop stand, and root development thereby improving the uptake of water and nutrients^4,28^. In the Western IGP, ZT enables early wheat seeding by about 2 weeks which along with residue mulch resulted in nullifying the ‘terminal heat effect’ during wheat grain filling^11,19,39^. The higher (by ~ 10%) system productivity (rice equivalent yield) with CA-based MW and RW system compared to CT-based system was reported previously by the other researchers in the region^10,11^. Further, mungbean integration also helps in improving the system productivity and profitability in similar ecologies^23,34^. The combined effect of mungbean into cereal (rice/maize) systems contributes towards the CA-based sustainable intensification in the IGP^10,11,23^.

Compared to the CT-based scenario, total production cost was ~ 13% lower under CA-based management systems when rice and maize was direct-seeded under ZT conditions. This was mainly due to reduction in tillage, puddling, and labour cost for manual transplanting in PTR. Similarly, ~ 24% higher cultivation cost was incurred in CT-MW system compared to CA-based management scenarios and it was due to the additional cost incurred in 3–4 tillage operations for preparing FB for maize planting and for irrigation. In addition, higher crop yields obtained in CA-based systems compared with CT also contributed towards the higher net returns (Fig. 1 and Table 2). Consistent with our study, Gathala et al*.*^38^ reported that the adoption of zero-till in DSR reduced the cost of cultivation by 79–85% compared to farmers’ practice of manual transplanting in rice. Due to the lower production cost, higher net incomes were associated with CA-based scenarios and our results are in consistent with the findings of Jat et al*.*^16,34^. Higher crop yields along with lower production costs in CA-based management practices resulted higher profitability compared with farmers’ practice as reported by many researchers^4,11,23,34^ in rice–wheat systems in IGP of India. Sustainable intensification of the CA-based MW system through mungbean integration provided the maximum net profit which was higher by USD 451 ha^−1^ compared to the CT-based scenario (Sc1) and was mainly due to additional income generated from mungbean.

CA-based management practices reduced irrigation water use by about 19% in rice and 77% in maize compared to Sc1 (Fig. 2). A similar saving of about 15–20% in DSR was reported by Kakraliya et al.^4^. The lower irrigation water use in rice under Sc2 and Sc3 was mainly due to avoidance of puddling which requires water equivalents to 3–4 irrigations and in combination with crop residues retention that probably minimized the evaporation loss from the soil surface. Application of water in maize was lowest with PBs due to lower water requirement and increased application efficiency^23,36,40^. In case of wheat, PBs reduced irrigation water by ~ 12% (4-years’ mean) compared to Sc1 (Fig. 2).The highest irrigation water productivity (WP_I_) was recorded with CA-based MW system (~ 2.24 kg grain m^−3^) followed by CA-based RW system (~ 0.51 kg grain m^−3^) compared to CT-based Sc1 (0.42 kg grain m^−3^). This was mainly due to less irrigation water used (Table 2) coupled with higher grain yields of rice, maize, and wheat (Table 1). Similar results of higher WP_I_ in CA-based RW and MW systems in the IGP of India were recorded by many researchers^21,23,34,40^. Higher values of WP_I_ in the MW system on PBs compared to flat planting were also reported by Jat et al*.*^41^.

The highest energy input together with the lowest energy output led to the lowest EUE under CT-based scenario (farmers’ practice). This was mainly due to more tillage, irrigation water, and labor and fertilizer inputs usage under the CT-based system^11,23^. In contrast, the combination of less input with higher energy output under CA-based management practices resulted in the highest EUE in rice, maize, and wheat crop. On system basis, the average EUE was 23 and 119% higher under CA-based rice (Sc2-Sc3) and maize (Sc5-Sc7) systems, respectively compared to CT-based Sc1 (Table 2). Kumar et al*.*^11^ and Kakraliya et al*.*^4^ reported that intensive tillage for seedbed preparation needed about one-third of the total operational energy that could be saved under ZT without adversely affecting the crop yields. The higher EUE was associated with lesser irrigation input (Table 2) in the CA-based MW system compared to the CA-based RW system. Jat et al*.*^41^ suggested that EUE was improved with reduction in tillage operations, and efficient water and nutrient management in MW system. CA along with efficient and precise use of inputs is conducive to optimizing the EUE in cereal based system in the IGP. Our results are in accordance with Kakraliya et al*.*^4^ and Jat et al*.*^16^.

CA-based management practices of rice had 39% (4-years’ mean) lower CH_4_ emissions than CT rice (Table 3). This was mainly due to anaerobic conditions caused by puddling and continuous flooding which are conducive to CH_4_ production and emission. Gupta et al*.*^42^ also highlighted that maintenance of intermittent wetting and drying conditions in DSR reduced CH_4_ emission by 30% over transplanted puddled rice (TPR). The aerobic zones in DSR keep the redox potential below the threshold level for the production of CH_4_^43^. Conventional puddled transplanting of rice stimulated CH_4_ emission from the soil, which was further increased with the incorporation of crop residues in the soil^7^. Seasonal N_2_O emission in rice in different treatments varied from 7 to 580 kg CO_2_ eq. ha^−1^, with a mean value of 390 kg CO_2_ eq. ha^−1^ (Table 3). Frequent wetting and drying of soil under DSR might lead to more emissions of N_2_O from the microbial nitrification–denitrification process in the soil. Higher N_2_O emission in DSR was also confirmed by Gupta et al*.*^42^ in the IGP region.In farmers’ practice, continuous submergence might have reduced nitrification process and thereby reduction in denitrification (conversion of NO_3_^−^ to N_2_). According to Gupta et al*.*^42^ another reason for the low level of N_2_O observed in the TPR could probably be due to the fast conversion rate of NO_3_^−^ to N_2_ through complete denitrification without forming N_2_O as an intermediate product. In wheat, the highest N_2_O emission was recorded with CA-based management practices and this was probably due to the greater availability of easily oxidizable C in CA plots that favored the process of denitrification of applied N under partial aerobic soil environments^42^. Kakraliya et al*.*^4^ and Kumar et al*.*^11^ also observed more N_2_O emission in ZT over CT-wheat from North-western IGP. On the system basis, CA-based management practices recorded lower energy-related emission of GHG by 34% over farmers’ practice. Intensive tillage and higher irrigation water use in farmers’ practice led to higher energy-related GHG emissions compared to CA-based scenarios, since the latter requires many fewer tractor hours. By adopting only ZT in wheat crop alone, IGP farmers could save about 36 L diesel ha^−1^ (Erenstein and Laxmi^44^) which is equivalent to 93 kg CO_2_ emission ha^−1^ year^−1^. CA-based practices can also mitigate GHG emissions by reducing pumping for irrigation^7^.

Higher C-sequestration under CA-based management practices than CT-based practices was due to least soil disturbance, retention/incorporation of crop residues, greater biomass input, and a lower rate of decomposition as reported by Sapkota et al*.*^12^. Zero-tillage minimizes the disruption of soil macro- and micro-aggregates which protects soil organic carbon (SOC) from microbial decomposition. They also reported higher C-sequestration in ZT than the CT-based RW system through seven years of experimentation in IGP. The significant management effects were recorded for GWP due to the variations in crops and management practices (tillage, crop establishment, residue retention, water management) and changes in SOC under different scenarios. The lower GWP under CA-based management scenarios might be due to the layering of best crop management practices that helped in mitigation of GHG emission. Consistent to the results from our study, Sapkota et al*.*^7^ and Gupta et al*.*^42^ also reported a reduction in GWP by 44–47% in the CA-based RW system without significant penalty in system yield compared to the CT-based system. A higher share of rice to total GWP than wheat was chiefly due to higher CH_4_ emission in rice and also higher energy consumption in rice for tillage and irrigation compared to wheat.

## Conclusions

A sound agronomic management practice portfolios (tillage, crop establishment, and residue management) related to crops and cropping system, can provide a potential option for sustaining the natural resources in Western IGP without sacrificing the systems productivity and farm profitability, and environmental quality. The CA-based management practices in both RW and MW systems remarkably enhances the response of other component technologies in terms of resources use efficiency (water and energy) while reducing environmental footprints compared to CT-based management practices. Among both the cereal systems, CA-based rice–wheat rotation from RW scenarios and CA-based maize-wheat-mungbean rotation from MW scenarios was found most efficient in terms of productivity (crop and water), profitability and environmental quality. The CA-based maize-wheat-mungbean system increased the system productivity by 11%, and profitability by 25% (USD 452 ha^−1^) with 64% less irrigation water while reducing the GWP by 106% compared to CT-based rice–wheat system (farmers’ practice). Compared to farmers’ practice of the RW system, the GWP was reduced by 99% with CA-based management practices in MW system. In western IGP, the rising cost of cultivation, declining profitability, and degradation of natural resources are the major drivers to seek the farmers for alternatives such as CA-based maize systems, which requires fewer resources and capital than traditional practices of rice systems.

## Methods

### Site characteristic

A field study was conducted for 4-years from 2014–15 to 2017–18 at ICAR (Indian Council of Agricultural Research)-CSSRI (Central Soil Salinity Research Institute) research platform, Karnal (29°42ʺ20.7ʹ N latitude, 76°57ʺ19.79ʹ E longitude, 243 m elevation), India. The region is characterized by a sub-tropical climate with wet summers and dry winters, with an average annual rainfall of 670 mm, 75–80% of which occurs from June to September (monsoon season). The climate has three distinct seasons i.e. wet/*kharif* (July–October), dry/r*abi* (November–March), and summer/*zaid* (April–June). The experimental soil was silty loam in texture, low in organic carbon (0.48%) and major nutrients (N, P, K) with a slightly alkaline pH (8.13). The initial soil characteristics of the experimental site are given in Table S5.

### Experimental details and scenarios description

Before imposing treatments in 2014, the experiment was laid out in a randomized complete block design with three replications in November, 2013 anda uniform wheat crop was planted as a cover crop in all the plots. The treatments consisted of seven scenarios (Sc) with different combinations of tillage and crop establishment practices, crop residue management, and cropping systems: Sc1-farmers’ practice-puddled transplanted rice (PTR) followed by (*fb*) conventional tillage (CT) wheat without residue (−R); Sc2-CT direct-seeded rice (DSR) *fb* Zero tillage (ZT) wheat with residue (+ R); Sc3-ZT direct seeded rice *fb* ZT wheat (+ R); Sc4-maize on fresh beds (FB) *fb* CT wheat (−R); Sc4-maize on permanent beds (PB) *fb* ZT wheat (+ R); Sc6-ZT maize *fb* ZT wheat (+ R); Sc7-ZT maize *fb* ZT wheat *fb* ZT mungbean (+ R) . The Sc2 to S3 and Sc5 to Sc7 were based on conservation agriculture (CA). CT-based rice–wheat system (Sc1) was considered as farmers’ practice as it is common in north-west India. The experiment was conducted in a plot size of 650 m^2^ where tractors can move freely for every operation. The description of different scenarios is provided in Table 4.

**Table 4:** Drivers of agricultural change, crop rotation, tillage, crop establishment method, and residue management under different scenarios.

Scenarios (Sc)	Drivers of change	Crop rotation	Tillage	Crop establishment	Residue management
1	Business as usual (Farmer’s practice)	Rice–Wheat-Fallow	Conventional tillage (CT) rice and wheat	Rice: transplanting Wheat: broadcast	All residue removed
2	Increase food production and income	Rice–wheat-Fallow	CT direct seeded rice (CTDSR) –Zero tillage (ZT) wheat	Rice:drill seeding Wheat: drill seeding	Full (100%) rice residue retained and wheat residue incorporated
3	Deal with rising scarcity of labor, water, energy, degrading soil health and emerging climatic variability	Rice–wheat-Fallow	ZTdirect seeded rice (ZTDSR)– ZT wheat	Rice: Drill seeding Wheat: Drill seeding	Full (100%) rice and anchored (15–20 cm height) wheat residue retained
4	Farmer’s practice for maize based system	Maize–wheat-Fallow	Maize- Fresh beds (FB);Wheat –CT	Maize: Drill seeding Wheat: Broadcast	All residues removed
5	Deal with rising scarcity of labor, water, energy, degrading soil health and emerging climatic variability	Maize–wheat-Fallow	Permanent beds (PB)	Same as in Sc3 using multi crop bed planter	Anchored residue of both the crops retained
6	Same as Sc5	Maize–wheat-Fallow	ZT in both the cropson flat beds	Same as in Sc3	Anchored residue of both the crops retained
7	Sustainable intensification of MW system through mungbean integration to deal same issues as in Sc3	Maize–Wheat–Mungbean	ZT in all the three cropson flat beds	Maize: Drill seeding Wheat: Drill seeding Mungbean: Drill/relay	Anchored residue of both rice and wheat and full mungbean residue retained

### Soil sampling and analysis

After harvesting of wheat (uniform crop) in 2014, soil samples were collected from 0–15 cm soil depths using an auger (5 cm internal diameter). Each plot was divided into four grids of 10 m × 05 m. A composite sample was prepared from six randomly selected sample points within a plot. The soil samples were ground to pass through a 2-mm sieve after air-drying and stored in a jar for further laboratory analysis for selected soil properties.

### Crop residue management and estimation of residue bio-mass recycling

All previous crop residues were removed manually before crop planning in CT-based scenarios Sc1 and Sc4, whereas in CA-based scenarios (Sc2, Sc3, Sc5, Sc6, and Sc7), crop residues were retained/incorporated as per the treatment protocol. In Sc2, all rice residues were retained on the soil surface at wheat sowing but anchored wheat residues (~ 30%) were incorporated in the soil by tillage operations for rice. However, in Sc3 all rice residue and anchored wheat residue were retained on the soil surface. In Sc5, Sc6 and Sc7, partial (~ 65%) maize residues and anchored wheat stubbles (∼ 30%) were retained. Similarly, in Sc7, all mungbean residues were retained at the soil surface and maize and wheat residue were managed as Sc5. The amount of crop residue recycled in each scenario after the harvest of each crop was assessed by sampling five rows with a length of 1.0 m from three locations in each plot. Crop residues were harvested manually from the soil surface, oven-dried till the constant weight occurred, and expressed on a dry weight basis per hectare. Over the 4-year (2014–2018), 39, 39, 38, 37, and 46 Mg ha^−1^ of crop residues were recycled (retained or incorporated) for Sc2, Sc3, Sc5, Sc6, and Sc7, respectively (Table S6).

### Fertilizer and weed management

Rice, maize, and wheat were fertilized with recommended dose of 150 kg N + 60 kg P + 60 kg K over the years. During the experiment, 22.5 kg ha^−1^ N and the whole of the P and K fertilizers (and 25 kg ZnSO_4_ ha^−1^ to wheat crop only) were applied as basal at seeding/transplanting time in the form of diammonium phosphate and muriate of potash, respectively, while remaining N was top dressed as urea in three equal splits at the early establishment, active tillering and panicle initiation stage in rice and at 20 and 45 days after seeding (DAS) and tasseling/silking (55–60 DAS) stage in maize. However, urea was top-dressed in two equal splits in wheat at crown root initiation (20–25 DAS) and maximum tillering stage (50–55 DAS). Crop management practices under different scenarios are given in Table 5.

**Table 5:** Crop management practices under different scenarios in rice/maize based cropping systems.

Scenarios^a^	Sc1	Sc2	Sc3	Sc4	Sc5	Sc6	Sc7
Field preparation	Rice- 2 pass of harrow, 1 pass of rotavator, 2 pass of puddle harrow followed by (fb) planking; Wheat- 2 pass of harrow and rotavator each fb planking	Rice-1 pass of harrow, 1 pass of cultivator fb planking; Wheat- Zero tillage	Direct sowing under ZT condition	Maize- 2 pass of harrow and rotavator each fb planking Wheat- 2 pass of harrow and rotavator each fb planking	Direct sowing on permanent beds	Direct sowing under ZT condition	Direct sowing under ZT condition
Seed rate (kg ha^−1^)^b^	Rice- 12.5; Wheat- 100	Rice- 20; Wheat- 100	Rice- 20; Wheat- 100	Maize- 20; Wheat- 100	Maize- 20; Wheat- 80	Maize- 20; Wheat- 100	Maize- 20; Wheat- 100; Mungbean-20
Equipment used for sowing	Rice- Manual transplanting Wheat- Manual broadcasting	Rice- Multi-crop planter Wheat- Happy seeder (HS)	Happy seeder in both the crops	Maize- Bed planter Wheat- Manual broadcasting	Maize- Bed planter Wheat- Bed planter	Happy seeder in both the crops	Happy seeder in all the crops
Crop geometry	Random geometry	22.5 cm–22.5 cm	22.5 cm–22.5 cm	67.5 cm–22.5 cm	67.5 cm–22.5 cm	67.5 cm–22.5 cm	67.5 cm–22.5 cm–22.5 cm
Fertilizer (N:P:K) in kg ha^−1^	Rice- 150:60:00 Wheat- 150:60:00 + ZnSO_4_ @25 kg ha^−1^	Rice- 150:60:60 Wheat- 150:60:60 + ZnSO_4_ @25 kg ha^−1^	Rice-150:60:60 Wheat- 150:60:60 + ZnSO_4_ @25 kg ha^−11^	Maize- 150:60:00 Wheat- 150:60:00 + ZnSO_4_ @25 kg ha^−1^	Maize- 150:60:60 Wheat- 150:60:60 + ZnSO_4_ @25 kg ha^−1^	Maize- 150:60:60 Wheat- 150:60:60 + ZnSO_4_ @25 kg ha^−1^	Maize- 150:60:60 Wheat- 150:60:60 + ZnSO_4_ @25 kg ha^−1^ Mungbean- 00:00:00
Water management	Rice- Continuous flooding of 5–6 cm depth for 30–40 days after transplanting fb irrigations applied at alternate wetting and drying Wheat- 4–6 irrigations as per requirement	Rice- Soil was kept wet up to 20 days after sowing fb irrigations applied at hair-line cracks Wheat- 4–6 irrigations as per critical crop growth stages	Same as in Sc2	Maize- 4–5 irrigations as per requirement Wheat- 5–7 irrigations as per requirement	Maize- 4–5 furrow irrigations as per requirement Wheat- 5–7 furrow irrigations as per requirement	Maize- 3–4 irrigations as per requirement Wheat- 4–6 irrigations as per requirement	Maize and wheat as Sc6 Mungbean- 1–2 irrigations as per need

^a^Refer Table 4 for scenarios description.

^b^Seed treatment was done with Bavistin + Streptocycline (10 + 1 g per 10 kg seed) for wheat and Raxil Tebuconazole 2DS (2% w/w ) at 0.2 g a.i. kg^−1^ seed for rice and maize.

For controlling weeds, glyphosate @ 1.25 kg active ingradient per hectare (kg a.i. ha^−1^) was applied prior to seeding of rice, maize, and wheat in PBs and ZT plots, however, no herbicides were applied in conventionally-till (CT) plots before sowing. The weeds were managed in all the scenarios by using pre- and post-emergence herbicides and one spot hand weeding as and when required. A spray of pendimethalin (1000 g a.i. ha^−1^) just one day after seeding as pre-emergence followed by bispyribac sodium (25 g a.i. ha^−1^) at 20–25 DAS as post-emergence herbicide was applied to control weeds in DSR in Sc2 and Sc3. In maize, atrazine (1000 g a.i. ha^−1^) and tembotrione (90 g a.i. ha^−1^) were applied as pre- and post-emergence, repectively. In wheat, a pre-mix combination of clodinafop ethyl + metsulfuron (60 + 4 g a.i. ha^−1^) was applied at 30–35 DAS.

### Crop data and economics

In all scenarios (Sc1-Sc3) rice was harvested and threshed either by combine harvester or manually at a height of 25–30 cm from ground level except Sc1 that was harvested at ground level. Maize was harvested or cob picked manually and threshed mechanically using a maize sheller. Wheat was harvested by either a combine harvester or a reaper binder at about 15–20 cm above ground level in all the CA-based scenarios except Sc1 and Sc4 where it was harvested at ground level. For wheat and rice, the grain and straw yields were determined on a total area of 99.0 m^2^ by sampling from four locations of 24.75 m^2^ each. For maize, yields (grain and straw) were measured on a total area of 108 m^2^ by sampling from four locations of 27 m^2^ each. The entire plot was harvested for mungbean yield estimation. The system-level yield/productivity of different cropping systems was estimated on rice equivalent basis in which the yield of wheat, maize, and mungbean was converted into rice equivalent yield (REY) (Mg ha^−1^) and calculated as follows the Eq. (1).

1 $$ {\text{Rice equivalent yield }} = {\text{Grain yield of non } - \text{ rice crop }}\left( {{\text{Mg ha}}^{{ - {1}}} } \right)*{\text{ MSP of non } - \text{ rice crop }}\left( {{\text{USD Mg}}^{{ - {1}}} } \right)/{\text{MSP of rice }}\left( {{\text{USD Mg}}^{{ - {1}}} } \right) $$

*where,* MSP is the Minimum Support Price (Table S2); (1 USD = 66.26 Indian Rupee).

The data on crop management inputs like tillage, irrigations, seed, pesticides, fertilizer, labor use, etc. and their costs under each scenario were recorded using a standard data format. All these variable costs for different scenarios were summed up to calculate the cost of production. The cost of key inputs and outputs during different years are presented in Table S7. Gross returns were calculated on the prevailing market prices of the produce (grain and straw) over the years (Table S7). Net returns were calculated by deducting the total cost of cultivation (Table S1) from the gross returns.

### Irrigation management

To calculate the irrigation water used, the water meter reading (kiloliter, kL) was recorded from each plot and presented as mm ha^−1^. The total rainfall was recorded using a rain gauge installed adjacent to the experimental field (Fig. S1). Water productivity for irrigation (WP_I_) was calculated by using Eq. (2).

2 $$ {\text{Irrigation water productivity Grain yield }}({\text{kg ha}}^{{ - {1}}} )/{\text{irrigationwater used }}({\text{mm ha}}^{{ - {1}}} ). $$

### Sustainable yield index (SYI)

Total crop productivity of rice, maize, and wheat was calculated through a SYI using yield-data of 4 years. This was done to adjust any seasonal/annual variations in the crop yield due to climatic condition and to highlight the relative productivity of the scenarios for the entire experimental period. The SYI is defined according to Eq. (3)

3 $$ {\text{SYI }} = \, \left( {{\text{Y}} - \, \sigma } \right)/{\text{Y}}_{{\max}} $$

*where,* Y is the estimated average yield of practice across the years, σ is its estimated standard deviation, and Y_max_ is the observed maximum yield in the experiment during the years of cultivation^45^.

### Energy analysis

The energy equivalent (MJ unit^−1^) of each input was used (as per Kakraliya et al*.*^4^, Table S2) to calculate the overall energy used in each crop under various scenarios. To estimate energy input, we considered all variable production inputs namely machinery, human labor, diesel, seed, fertilizer, irrigation, pesticides etc. and for energy outputs, total crop biomass (grain and straw) were considered. Based on the energy equivalents of the inputs (Table S3) and outputs (Table S4), energy use efficiency (Fig. 4 and Table 2) and specific energy were calculated using Eqs. (4) and (5).

4 $$ {\text{Energy use efficiency }} = {\text{ Total energy Output }}\left( {{\text{MJ ha}}^{{ - {1}}} } \right)/{\text{Total energy Input }}\left( {{\text{MJ ha}}^{{ - {1}}} } \right) $$

5 $$ {\text{Specific energy }}\left( {{\text{MJ kg}}^{{ - {1}}} } \right) \, = {\text{ Total energy input }}\left( {{\text{MJ ha}}^{{ - {1}}} } \right)/{\text{ Grain output }}\left( {{\text{kg ha}}^{{ - {1}}} } \right) $$

### Global warming potential (GWP) analysis

Net GWP of rice, maize, wheat, and cropping systems was estimated by using all the sources and sinks of greenhouse gases (GHGs) such as emissions due to production and transportation of fertilizers, field operations (tillage, seeding, irrigation), retention/incorporation of crop residues, land use management, C-sequestration and soil flux of GHGs. The emissions of GHGs were computed by using the CCAFS Mitigation Option Tool (CCAFS-MOT^46^). In this tool, many empirical models are combined to compute GHG emissions in any production system. The tool considers specific factors namely: climatic conditions, soil characteristics, crop production inputs, and other management activities that influence emissions. The background and fertilizer-induced emissions are estimated using the multivariate empirical model (MEM) of Bouwman and Boumans^47^ for nitrous oxide (N_2_O), and nitric oxide (NO) emissions, and FAO/IFA^48^ model for ammonia (NH_3_) emission. Emissions led by crop residues were computed through IPCC N_2_O Tier-1 emission factors. Alike, the Ecoinvent database was used for emission released from the crop production and fertilizer transportation^49^. Alterations in SOC due to tillage operations, farmyard manure, and residue retention/incorporation are based on IPCC methodology as described by Smith et al*.*^50^ (1997) and Ogle et al*.*^51^. The CO_2_ emissions from soil resulting from urea or liming were calculated as projected by IPCC methodology (IPCC, 2006). GWP of the different production systems/scenarios were computed on base GWP (over 100 years) of 298 for N_2_O and 34 for CH_4_ (IPCC^52^). Global warming potential (GWP) and total GWP were calculated using Eqs. (6) and (7).

6 $$ {\text{GWP }}\left( {{\text{kg CO}}_{{2}} {\text{eq}}./{\text{ha}}} \right) \, = {\text{ CO}}_{{2}} \left( {{\text{kg}}/{\text{ha}}} \right) \, + {\text{ N}}_{{2}} {\text{O }}\left( {{\text{kg}}/{\text{ha}}} \right) \, \times { 298 } + {\text{ CH}}_{{4}} \left( {{\text{kg}}/{\text{ha}}} \right) \, \times { 34}) $$

7 $$ {\text{Total GWP }} = \, \Delta {\text{soil C GWP } + \text{ soil CH}}_{{4}} {\text{emission }} + {\text{ soil N}}_{{2}} {\text{O emission }} + {\text{ operation GHG emission }} + {\text{ input GHG emission}} $$

### Statistical analysis

Analysis of variance for randomized complete block design was performed using the general linear model procedures of the statistical analysis system (SAS Institute, Cary, NC). The differences between treatment means were compared using Tukey’s HSD test at P < 0.05^53^.

## Supplementary information

Supplementary Information 1.

Supplementary Information 2.

## Abbreviations

C: Carbon
CA: Conservation agriculture
CH_4_: Methane
CO_2_: Carbon dioxide
CT: Conventional tillage
DAS: Days after sowing
DSR: Direct seeded rice
EUE: Energy use efficiency
FB: Fresh beds
FP: Farmers’ practice
GHG: Greenhouse gas
GWP: Global warming potential
IGP: Indo-gnagetic plains
IPCC: Intergovernmental panel on climate change
K: Potassium
Mg: Mega gram
MJ: Mega joule
MW: Maize-wheat
N: Nitrogen
N_2_O: Nitrous oxide
NO: Nitric oxide
NW: North-west
P: Phosphorus
PB: Permanent beds
PTR: Puddled transplanted rice
REY: Rice equivalent yield
RW: Rice–wheat
SAS: Statistical analysis system
SOC: Soil organic carbon
SYI: Sustainable yield index
TPRp: Transplanted puddled rice
USD: United states dollar
WP_I_: Irrigation water productivity
ZT: Zero tillage
