# Supplementary material for: Designing profitable, resource use efficient and environmentally sound cereal based systems for the Western Indo-Gangetic plains
Source: Sci Rep. 2020 Nov 6;10:19267. doi: 10.1038/s41598-020-76035-z (PMC7648623; doi:10.1038/s41598-020-76035-z)
Supplement: Supplementary file 1 — Supplementary Information 1. [file 41598_2020_76035_MOESM1_ESM.docx]

**Designing profitable, resource use efficient and environmentally sound cereal-based systems for Western Indo-Gangetic plains**

Hanuman S. Jat^1,2*^, Virender Kumar^3^, Ashim Datta^1^, Madhu Choudhary^1^, Yadvinder-Singh^4^, Suresh K. Kakraliya^1^, Tanuja Poonia^5^, Andrew McDonald^6^, Mangi L. Jat^2^ and Parbodh C. Sharma^1*^

^1^ICAR-Central Soil Salinity Research Institute (CSSRI), Karnal, India

^2^International Maize and Wheat Improvement Center (CIMMYT), New Delhi, India

^3^International Rice Research Institute (IRRI), Los Banos, Philippines

^4^Borlaug Institute for South Asia (BISA), Ludhiana, India

^5^Swami Keshwanand Rajasthan Agriculture University, Bikaner, India

^6^Collage of Agriculture and Plant Sciences, Cornell University, Ithaca NY 14853

*Corresponding Authors:

Dr. Hanuman S. Jat, Principal Scientist, ICAR-CSSRI, Karnal

**Tel:** +91 (184) 2290501; Mob**:** +91 9050002757

Email: hsjat_agron@yahoo.com

Dr. Parbodh C. Sharma, Director, ICAR-CSSRI, Karnal

**Tel:** +91 (184) 2290501; Mob**:** +91 9416296240

Email: [pcsharma.knl@gmail.com](mailto:pcsharma.knl@gmail.com)

**Fig. S1**

Annual (2014–15, 2015–16, 2016–17 and 2017-18) and long-term (1981–2018) weather data related to rainfall and temperature (maximum and minimum).

**Fig. S2**

Effect of different scenarios on sustainable yield index (SYI) of crops and cropping systems.
