# Supplementary material for: Designing profitable, resource use efficient and environmentally sound cereal based systems for the Western Indo-Gangetic plains
Source: Sci Rep. 2020 Nov 6;10:19267. doi: 10.1038/s41598-020-76035-z (PMC7648623; doi:10.1038/s41598-020-76035-z)
Supplement: Supplementary file 2 — Supplementary Information 2. [file 41598_2020_76035_MOESM2_ESM.docx]

**Designing profitable, resource use efficient and environmentally sound cereal-based systems for Western Indo-Gangetic plains**

Hanuman S. Jat^1,2*^, Virender Kumar^3^, Ashim Datta^1^, Madhu Choudhary^1^, Yadvinder-Singh^4^, Suresh K. Kakraliya^1^, Tanuja Poonia^5^, Andrew McDonald^6^, Mangi L. Jat^2^ and Parbodh C. Sharma^1*^

^1^ICAR-Central Soil Salinity Research Institute (CSSRI), Karnal, India

^2^International Maize and Wheat Improvement Center (CIMMYT), New Delhi, India

^3^International Rice Research Institute (IRRI), Los Banos, Philippines

^4^Borlaug Institute for South Asia (BISA), Ludhiana, India

^5^Swami Keshwanand Rajasthan Agriculture University, Bikaner, India

^6^Collage of Agriculture and Plant Sciences, Cornell University, Ithaca NY 14853

*Corresponding Authors:

Dr. Hanuman S. Jat, Principal Scientist, ICAR-CSSRI, Karnal

**Tel:** +91 (184) 2290501; Mob**:** +91 9050002757

Email: hsjat_agron@yahoo.com

Dr. Parbodh C. Sharma, Director, ICAR-CSSRI, Karnal

**Tel:** +91 (184) 2290501; Mob**:** +91 9416296240

Email: [pcsharma.knl@gmail.com](mailto:pcsharma.knl@gmail.com)

**Table S1**

Effect of different management practices on cost of cultivation (USD ha^-1^) of rice, maize, wheat and systems under different scenarios in 4 years (2014-18)

| Scenarios^a^ | 2014-15 | | | 2015-16 | | | 2016-17 | | | 2017-18 | | | 4-yrs’ mean | | |
| --- | --- | --- | --- | --- | --- | --- | --- | --- | --- | --- | --- | --- | --- | --- | --- |
|  | Rice/ Maize | Wheat | System | Rice/ Maize | Wheat | System | Rice/ Maize | Wheat | System | Rice/ Maize | Wheat | System | Rice/ Maize | Wheat | System |
| Sc1 | 715^Ab^ | 465^A^ | 1180^A^ | 626^A^ | 529^A^ | 1155^A^ | 670^A^ | 559^A^ | 1229^A^ | 707^A^ | 582^A^ | 1290^A^ | 680^A^ | 534^A^ | 1213^A^ |
| Sc2 | 677^B^ | 397^B^ | 1074^B^ | 581^B^ | 457^B^ | 1037^CD^ | 567^D^ | 479^B^ | 1046^CD^ | 629^C^ | 499^B^ | 1128^C^ | 613^C^ | 458^B^ | 1071^BC^ |
| Sc3 | 643^C^ | 395^B^ | 1038^BC^ | 561^C^ | 458^B^ | 1019^D^ | 541^E^ | 479^B^ | 1020^D^ | 601^E^ | 500^B^ | 1101^C^ | 586^DE^ | 458^B^ | 1044^C^ |
| Sc4 | 629^C^ | 432^AB^ | 1061^B^ | 586^B^ | 491^AB^ | 1076^BC^ | 631^B^ | 516^AB^ | 1148^B^ | 673^B^ | 539^AB^ | 1212^B^ | 630^B^ | 495^AB^ | 1124^B^ |
| Sc5 | 582^D^ | 410^AB^ | 992^C^ | 545^D^ | 470^B^ | 1015^D^ | 587^C^ | 495^B^ | 1083^C^ | 616^D^ | 515^B^ | 1131^C^ | 583^E^ | 473^B^ | 1055^C^ |
| Sc6 | 595^D^ | 393^B^ | 988^C^ | 549^D^ | 458^B^ | 1007^D^ | 593^C^ | 477^B^ | 1070^CD^ | 631^C^ | 497^B^ | 1128^C^ | 592^D^ | 456^B^ | 1048^C^ |
| Sc7 | 593^D^ | 396^B^ | 1135^A^ | 549^D^ | 459^B^ | 1125^AB^ | 594^C^ | 479^B^ | 1193^AB^ | 635^C^ | 501^B^ | 1282^A^ | 593^D^ | 459^B^ | 1184^A^ |

^a^Refer Table 4 for scenarios description

^b^Means followed by a similar uppercase letters within a column are not significantly different at 0.05 level of probability using Tukey’s HSD test.

**Table S2**

Energy equivalents used in the study for different agricultural operations

| Particulars | Units | Unit energy equivalent (MJ Unit^-1^) | References |
| --- | --- | --- | --- |
| *Input* |  |  |  |
| Human labour | Man-hour | 1.96 | Gathala *et al.*^54^ |
| Diesel | Litre | 56.31 | Gathala *et al.*^54^ |
| Petrol | Litre | 46.30 |  |
| Nitrogen (N) | kg | 66.14 | Gathala *et al.*^54^ |
| Phosphorus (P_2_O_5_) | kg | 22.44 | Gathala *et al.*^54^ |
| Potassium (K_2_O) | kg | 11.15 | Gathala *et al.*^54^ |
| Herbicides, insecticides and pesticides | kg | 120.00 | Gathala *et al.*^54^ |
| Irrigation water | ha-cm | 143.56 | Gathala *et al.*^54^ |
| Zinc sulphate (ZnSO_4_) | kg | 8.40 | Argiro *et al.*^55^ |
| Iron sulphate (FeSO_4_) | kg | 110.00 | Argiro *et al.*^55^ |
| Rice /Wheat/ Maize/Mungbean seed | kg | 14.70 | Ozkan *et al.*^56^ |
| Tractor | kg | 93.61 |  |
| Other machinery | kg | 62.70 |  |
| Combine harvester | kg | 87.63 |  |
| *Output* |  |  |  |
| Rice /Wheat/Maize/Mungbean grain | kg | 14.70 | Ozkan *et al.*^56^ |
| Rice/Wheat/Maize/Mungbean Straw | kg | 12.50 | Ozkan *et al.*^56^ |

**Table S3**

Effect of different scenarios on energy input (10^3^ MJ ha^-1^) of rice, maize, wheat and systems in 4 years (2014-18)

| Scenarios^a^ | 2014-15 | | | 2015-16 | | | 2016-17 | | | 2017-18 | | | 4-yrs’ mean | | |
| --- | --- | --- | --- | --- | --- | --- | --- | --- | --- | --- | --- | --- | --- | --- | --- |
|  | Rice/ Maize | Wheat | System | Rice/ Maize | Wheat | System | Rice/ Maize | Wheat | System | Rice/ Maize | Wheat | System | Rice/ Maize | Wheat | System |
| Sc1 | 50.64 | 22.94 | 73.59 | 48.45 | 24.86 | 73.31 | 58.58 | 24.70 | 83.29 | 55.49 | 24.80 | 80.29 | 53.29 | 24.33 | 77.62 |
| Sc2 | 45.31 | 19.18 | 64.49 | 38.10 | 21.11 | 59.21 | 47.83 | 20.95 | 68.79 | 50.57 | 20.87 | 71.44 | 45.45 | 20.53 | 65.98 |
| Sc3 | 42.51 | 18.83 | 61.34 | 37.56 | 21.37 | 58.93 | 46.22 | 20.95 | 67.17 | 48.88 | 21.09 | 69.97 | 43.79 | 20.56 | 64.35 |
| Sc4 | 22.62 | 21.81 | 44.43 | 20.94 | 23.47 | 44.41 | 19.58 | 23.45 | 43.03 | 24.17 | 23.60 | 47.77 | 21.83 | 23.08 | 44.91 |
| Sc5 | 20.51 | 18.10 | 38.61 | 19.54 | 20.38 | 39.91 | 17.65 | 19.96 | 37.61 | 21.55 | 19.87 | 41.42 | 19.81 | 19.58 | 39.39 |
| Sc6 | 22.66 | 18.76 | 41.42 | 19.77 | 21.39 | 41.17 | 18.24 | 21.24 | 39.48 | 23.56 | 21.34 | 44.89 | 21.06 | 20.68 | 41.74 |
| Sc7 | 22.30 | 19.11 | 45.08 | 19.69 | 21.39 | 44.73 | 18.38 | 20.84 | 43.07 | 24.22 | 21.21 | 49.28 | 21.15 | 20.64 | 45.54 |

^a^Refer Table4 for scenarios description

**Table S4**

Effect of different scenarios on energy output (10^3^ MJ ha^-1^) of rice, maize, wheat and systems in 4 years (2014-18)

| Scenarios^a^ | 2014-15 | | | 2015-16 | | | 2016-17 | | | 2017-18 | | | 4-yrs’ mean | | |
| --- | --- | --- | --- | --- | --- | --- | --- | --- | --- | --- | --- | --- | --- | --- | --- |
|  | Rice/ Maize | Wheat | System | Rice/ Maize | Wheat | System | Rice/ Maize | Wheat | System | Rice/ Maize | Wheat | System | Rice/ Maize | Wheat | System |
| Sc1 | 222 | 178 | 399 | 181 | 178 | 358 | 223 | 185 | 407 | 216 | 184 | 400 | 210 | 181 | 391 |
| Sc2 | 238 | 187 | 425 | 209 | 195 | 404 | 198 | 211 | 409 | 199 | 199 | 398 | 211 | 198 | 409 |
| Sc3 | 255 | 186 | 441 | 192 | 175 | 367 | 190 | 203 | 393 | 204 | 196 | 400 | 210 | 190 | 400 |
| Sc4 | 232 | 185 | 417 | 208 | 158 | 365 | 260 | 193 | 452 | 237 | 187 | 424 | 234 | 181 | 415 |
| Sc5 | 263 | 172 | 434 | 234 | 199 | 433 | 300 | 210 | 509 | 290 | 207 | 498 | 272 | 197 | 469 |
| Sc6 | 262 | 180 | 442 | 243 | 180 | 423 | 266 | 204 | 471 | 288 | 202 | 490 | 265 | 191 | 456 |
| Sc7 | 244 | 182 | 431 | 245 | 189 | 438 | 295 | 214 | 514 | 276 | 200 | 480 | 265 | 196 | 466 |

^a^Refer Table 4 for scenarios description

**Table S5**

Initial soil (0-15 cm) properties of the experimental field in 2014-15

| Properties | Value | Method Used |
| --- | --- | --- |
| Sand (%) | 45.20±0.32 | Particle size analysis^57^ |
| Silt (%) | 33.35±0.29 |  |
| Clay (%) | 21.45±0.12 |  |
| Textural class | Silty loam | USDA triangle |
| Bulk density (Mg m ^-3^) | 1.45±0.01 | Blake and Hartage^58^ |
| Infiltration rate (cm ha^-1^) | 0.39±0.04 | Double ring infiltrometer method^59^ |
| pH (1:2 soil: water) | 8.13±0.20 | Glass electrode pH meter^60^ |
| EC (dSm^-1^) | 0.23±0.05 | Conductivity bridge^60^ |
| Organic carbon (g kg^-1^) | 4.80±0.03 | Wet digestion method^61^ |
| Available P (mg kg^-1^) | 5.74 ±0.20 | 0.5 *M* NaHCO_3_ extractable^62^ |
| Available N (mg kg^-1^) | 120.21±3.15 | Alkaline permanganate method^63^ |
| 1 *M* Neutral NH4OAc- extractable K (mg kg^-1^) | 230.62±2.30 | Helmke and Sparks^64^ |

**Table S6**

Total residue load (Mg ha^-1^) in different crops under different scenarios over the years

| Scenarios^a^ | Residue incorporated/retained (Mg ha^-1^) | | | | | | | | | | | | | | | | |
| --- | --- | --- | --- | --- | --- | --- | --- | --- | --- | --- | --- | --- | --- | --- | --- | --- | --- |
|  | 2014-15 | | | | 2015-16 | | | | 2016-17 | | | | 2017-18 | | | | Grand total over 4 years |
|  | Rice/maize | Wheat | Mungbean | System | Rice/maize | Wheat | Mungbean | System | Rice/maize | Wheat | Mungbean | System | Rice/maize | Wheat | Mungbean | System |  |
| Sc1 | -NA^b^- | -NA- | -NA- | -NA- | -NA- | -NA- | -NA- | -NA- | -NA- | -NA- | -NA- | -NA- | -NA- | -NA- | -NA- | -NA- | -NA- |
| Sc2 | 8.85 | 1.80 | -NA- | 10.65 | 7.78 | 1.87 | -NA- | 9.65 | 7.38 | 2.03 | -NA- | 9.42 | 7.42 | 1.92 | -NA- | 9.34 | 39.1 |
| Sc3 | 9.65 | 1.79 | -NA- | 11.43 | 7.28 | 1.68 | -NA- | 8.96 | 7.19 | 1.95 | -NA- | 9.14 | 7.76 | 1.89 | -NA- | 9.65 | 39.2 |
| Sc4 | -NA- | -NA- | -NA- | -NA- | -NA- | -NA- | -NA- | -NA- | -NA- | -NA- | -NA- | -NA- | -NA- | -NA- | -NA- | -NA- | NA- |
| Sc5 | 3.97 | 1.66 | -NA- | 5.63 | 3.55 | 1.93 | -NA- | 5.47 | 4.53 | 2.02 | -NA- | 6.55 | 4.40 | 2.00 | -NA- | 6.40 | 38.2 |
| Sc6 | 3.98 | 1.73 | -NA- | 5.70 | 3.68 | 1.73 | -NA- | 5.41 | 4.03 | 1.97 | -NA- | 5.99 | 4.36 | 1.94 | -NA- | 6.31 | 37.2 |
| Sc7 | 4.26 | 1.75 | 2.25 | 8.27 | 3.70 | 1.82 | 1.95 | 7.47 | 4.47 | 2.06 | 2.11 | 8.63 | 4.20 | 1.93 | 2.09 | 8.22 | 46.8 |

^a^Refer Table 4 for scenarios description

^b^Not applicable

**Table S7**

Cost of key inputs and outputs used for economic analysis during the different years

| Item/Commodity | Cost (Indian National Rupee; INR) | | | |
| --- | --- | --- | --- | --- |
|  | 2014-15 | 2015-16 | 2016-17 | 2017-18 |
| Rice grain (kg^-1^) | 13.60 | 14.10 | 14.70 | 15.10 |
| Rice straw (kg^-1^) | -NA^a^- | -NA- | -NA- | -NA- |
| Rice seed (kg^-1^) | 260 | 280 | 300 | 350 |
| Maize grain (kg^-1^) | 13.10 | 13.25 | 13.65 | 13.65 |
| Maize straw (kg^-1^) | 1.20 | 1.20 | 1.80 | 1.80 |
| Maize seed (kg^-1^) | 260 | 280 | 300.0 | 300 |
| Wheat grain (kg^-1^) | 14.50 | 15.25 | 16.25 | 17.35 |
| Wheat straw (kg^-1^) | 2.3 | 2.8 | 3.00 | 3.00 |
| Wheat seed (kg^-1^) HD2967 | 28.00 | 28.00 | 32.00 | 32.00 |
| Mungbean grain (kg^-1^) | 46.00 | 48.50 | 52.25 | 55.75 |
| Mungbean straw (kg^-1^) | -NA- | -NA- | -NA- | -NA- |
| Mungbean seed (kg^-1^) SML 668 | 100.00 | 125.00 | 125.00 | 150.00 |
| Urea (kg^-1^) | 5.50 | 5.50 | 5.60 | 5.60 |
| Di-ammonium-phosphate (DAP) (kg^-1^) | 16.50 | 16.50 | 17.00 | 17.00 |
| Muriate of potash (MOP) (kg^-1^) | 22.50 | 22.50 | 23.2 | 23.2 |
| NPK Complex (kg^-1^) | 20.50 | 20.50 | 21.70 | 21.70 |
| Zinc sulphate (ZnSO_4_) (kg^-1^) | 35.00 | 35.00 | 35.70 | 35.70 |
| Diesel (l^-1^) | 54.50 | 55.70 | 55.23 | 60.00 |
| Wages Rate (person^-1^ day^-1^) | 300 | 350 | 350 | 360 |
| USD conversion rate | 66.26 | 66.26 | 66.26 | 66.26 |

^a^Not applicable

**References**

1. Gathala, M. K. *et al*. Productivity, profitability, and energetics: a multi-criteria assessment of farmers’ tillage and crop establishment options for maize in intensively cultivated environments of South Asia. *Field Crops Res*. **186**, 32–46 (2016).
2. Argiro, V., Strapatsa, A., George, D., Nanos, A. & Constantinos, A. Energy flow for integrated apple production in Greece. *Agric. Ecosyst. Environ*. **116**, 176–180 (2006).
3. Ozkan, B., Akcaoz, H. & Fert, C. Energy requirement input-output analysis in Turkish agriculture. *Renew. Energy***29**, 39–51 (2004).
4. Gee, G. W. & Baude, J. W. Particle-size analysis. In: A Klute, D.L., Campbell, G.S., Nielsen, D.R., Jackson, R.D., Mortland, M.M. (Eds.), Methods of Soil Analysis. Part 1. Physical and Mineralogical Methods. SSSA Book Series No. 9 (Part 1). ASA, SSSA, Madison, WI, pp. 383–411 (2006).
5. Blake, G. R. & Hartge, K. H. Bulk density. In: Klute, A. (Ed.), Methods of Soil Analysis. Part 1. Physical and Mineralogical Methods. American Society of Agronomy, Soil Science Society of America, Madison, WI, USA, pp. 363–375 (1986).
6. Bouwer, H. Intake rate: cylinder infiltrometer. In: method of soil analysis, part 1, physical and mineralogical properties. In: Klute, A. (Ed.), Agronomy Monograph No. 9. American Society of Agronomy Madison, Wisconsin, USA, pp. 825–843 (1986).
7. Richards, L. A. Diagnosis and improvement in saline, alkali soils. Handbook No. 60. USDA, Washington (1954).
8. Walkley, A. & Black, C. A. An examination of the method for determination of soil organic matter and proposed medication at the chromic acid titration method. *Soil Sci*. **37**, 29–38 (1934).
9. Olsen, B. C., Cole, C. V., Watenabe, F. S. & Dean, L. A. Estimation of Available Phosphorus by Extraction with Sodium Carbonate. *USDA Circular No. 939*, 19 (1954).
10. Subbiah, B. V. & Asija, G. L. A rapid procedure for the estimation of available nitrogen in soils. *Curr. Sci*. **25**, 259–260 (1956).
11. Helmke, P. A. & Sparks, D. L. Lithium, sodium, potassium, rubidium, and cesium. Methods of Soil Analysis: Part 3 Chemical Methods. **5**, 551-574 (1996).
